# Flow-sensitive four-dimensional magnetic resonance imaging facilitates the quantitative analysis of systemic-to-pulmonary collateral flow in patients with univentricular hearts

**DOI:** 10.1186/1532-429X-14-S1-W7

**Published:** 2012-02-01

**Authors:** Sarah Nordmeyer, Israel Valverde, Sergio Uribe, Gerald F Greil, Felix Berger, Titus Kuehne, Philipp B Beerbaum

**Affiliations:** 1 Department of Congenital Heart Disease/Pediatric Cardiology, Deutsches Herzzentrum Berlin, Berlin, Germany; 2 Pediatric Cardiology, King's College London, Division of Imaging Sciences, London, UK; 3 Radiology and Biomedical Imaging Center, Pontificia Universidad Catolica de Chile, Santiago, Chile

## Background

Systemic-to-pulmonary collateral flow (SPCF) may constitute a risk factor for increased morbidity and mortality in patients with single-ventricle physiology (SV). However, clinical research is limited by the complexity of multi-site two-dimensional (2D) cardiovascular magnetic resonance (CMR) flow assessment. We sought to validate four-dimensional flow (4D-flow) for concise quantification of SPCF in patients with SV.

## Methods

29 patients with SV physiology prospectively underwent CMR (1.5T) to quantify SPCF (n=14 bidirectional cavopulmonary connection [BCPC], age 2.9±1.3 years; and n=15 Fontan, 14.4±5.9 years) and 20 healthy volunteers (age, 28.7±13.1 years) served as controls. Five 2D-flow measurements (ascending aorta, superior/inferior caval veins, right/left pulmonary arteries) were performed and SPCF (=aortic minus caval flows) was calculated and compared with 4D-flow measurements and calculations. Additionally, 4D-flow measurements were used to calculate SPCF as pulmonary venous minus pulmonary arterial flow.

## Results

The comparison between 4D-flow and 2D-flow showed good Bland-Altman agreement for all individual vessels (mean bias, 0.05±0.24 l/min/m2), calculated SPCF (-0.02±0.18 l/min/m2), low intra and inter-observer variance (ICC>0.95[0.91-0.97]) and significantly shorter 4D-flow acquisition-time (12:34min/17:28min,p<0.01). 4D-flow in patients versus controls revealed (1) good agreement between systemic versus pulmonary estimator for SPFC; (2) significant SPCF in patients (BCPC 0.79±0.45 l/min/m2; Fontan 0.62±0.82 l/min/m2) and not in controls (0.01+0.16 l/min/m2) and (3) inverse relation of right/left pulmonary artery perfusion and right/left SPCF (Pearson=-0.47,p=0.01).

## Conclusions

4D-flow is reliable, operator-independent and more time-efficient than 2D-flow to quantify SPCF. There is considerable SPCF in BCPC and Fontan patients. SPCF was more pronounced towards the respective lung with less pulmonary arterial flow suggesting more collateral flow where less anterograde branch pulmonary artery perfusion.

